# Brown Adipose Tissue Heterogeneity, Energy Metabolism, and Beyond

**DOI:** 10.3389/fendo.2021.651763

**Published:** 2021-04-19

**Authors:** Abhijit Babaji Shinde, Anying Song, Qiong A. Wang

**Affiliations:** ^1^ Department of Molecular & Cellular Endocrinology, Arthur Riggs Diabetes and Metabolism Research Institute, Beckman Research Institute, City of Hope Medical Center, Duarte, CA, United States; ^2^ Comprehensive Cancer Center, Beckman Research Institute, City of Hope Medical Center, Duarte, CA, United States

**Keywords:** brown adipocyte development, brown adipocyte heterogeneity, thermogenesis, brown adipocyte energy metabolism, obesity

## Abstract

Brown adipocyte in brown adipose tissue (BAT) specializes in expending energy through non-shivering thermogenesis, a process that produces heat either by uncoupling protein 1 (UCP1) dependent uncoupling of mitochondrial respiration or by UCP1 independent mechanisms. Apart from this, there is ample evidence suggesting that BAT has an endocrine function. Studies in rodents point toward its vital roles in glucose and lipid homeostasis, making it an important therapeutic target for treating metabolic disorders related to morbidities such as obesity and type 2 diabetes. The rediscovery of thermogenically active BAT depots in humans by several independent research groups in the last decade has revitalized interest in BAT as an even more promising therapeutic intervention. Over the last few years, there has been overwhelming interest in understanding brown adipocyte’s developmental lineages and how brown adipocyte uniquely utilizes energy beyond UCP1 mediated uncoupling respiration. These new discoveries would be leveraged for designing novel therapeutic interventions for metabolic disorders.

## Introduction

Adipose tissue, one of the most plastic organs, is now widely accepted as an essential player in maintaining whole-body energy homeostasis (1, 2). Three different types of adipocytes that exist in mammals are white adipocytes, brown adipocytes, and beige or brite (stands for brown in white) adipocytes (3–6). Brown adipocytes and white adipocytes form a major part of brown adipose tissue (BAT) and white adipose tissue (WAT), respectively. In these adipose tissues, in addition to adipocytes, there are also stem cells, preadipocytes (committed adipocyte precursors), immune cells, fibroblasts, and endothelial cells. Both BAT and WAT appear as several discrete depots located throughout the body (7). Metabolically, WAT specializes in storing energy in the form of triglycerides. BAT expends energy *via* non-shivering thermogenesis, a process that involves dissipation of heat generated *via* uncoupling of mitochondrial respiration mediated by uncoupling protein 1 (UCP1) (8, 9). Morphologically, white adipocytes have unilocular lipid droplets, fewer mitochondria, and no expression of UCP1. Brown adipocytes have multilocular lipid droplets, high mitochondria content, and high expression of UCP1 (3–5). We recently showed that there are two subpopulations of brown adipocytes which co-exist in the BAT of mice (10). One subpopulation has high thermogenic activity with high UCP1 expression, and the other one has low thermogenic activity with low UCP1 expression. Conversely, beige adipocytes usually appear in the WAT depots in response to external cues such as cold exposure, exercise, or adrenergic stimulation. Although similar to white adipocytes they have low UCP1 expression, with external stimuli they can be activated to increase both UCP1 expression as well as respiration rate. Furthermore, they show a molecular signature that is distinct from either white or brown adipocytes (11, 12).

It is now evident that heterogeneity exists within the thermogenic adipocytes in both rodents and humans. Studying the developmental lineages of this heterogeneous cell population along with the investigation of the key mechanisms involved in the activation and thermoregulation of these adipocytes will enable us to identify novel therapeutic targets to treat metabolic disorders. In this review, we will discuss the developmental origins of BAT along with its heterogeneity followed by its developmental timeline. We will also summarize the recent works on BAT metabolism and fuel selection.

## Developmental Origins and Heterogeneity of BAT

Early lineage tracing studies suggested that brown adipocytes are closer to skeletal muscles in developmental origin than white adipocytes. Atit et al. showed that cells from mouse embryo central dermomyotome, which express the homeobox transcription factor Engrailed 1 at E9.5 give rise to interscapular BAT (iBAT), dermis, and skeletal muscles (13). After this observation, several other studies were published supporting the same notion that BAT and skeletal muscles share the common progenitors. Using a *Myf5^Cre^* driver crossed to a cytoplasmic reporter, the Spiegelman group elegantly showed that Myf5^+^ cells contribute to iBAT and skeletal muscles but not to any WAT depots (14). They also demonstrated that PR domain zinc-finger protein 16 (PRDM16) acts as a molecular switch between myoblast and brown fat cell lineages (14). Another study using an inducible Cre under the control of the *Pax7* promoter also showed that Pax7^+^ progenitors labeled at E9.5 give rise to iBAT. They also found that the myogenic restriction of the Pax7^+^ lineage occurs at the later stage in between E9.5 and E12.5 (15). Also, microarray analysis demonstrated that brown preadipocytes show myogenic transcription signature (16). Furthermore, when compared at transcriptome as well as proteome levels, brown fat mitochondria were found to share many similarities with muscle mitochondria (17). Remarkably, several factors including Ewing Sarcoma (EWS), Zinc Finger Protein 516 (ZFP516), Euchromatic Histone Lysine Methyltransferase 1 (EHMT1), early B cell factor-2 (EBF2), TATA-Box Binding Protein Associated Factor 7 Like (TAF7L), and some microRNAs (MyomiR-133, Mir193b-365) have been shown to affect cell fate decision between brown fat and muscle (14, 18–25). Using *Myf5^Cre^* crossed to *R26R-mTmG* reporter, the Guertin group suggested that brown adipocytes are not exclusively derived from *Myf5* lineage, and this lineage also contributes to white and beige adipocyte populations (26, 27). They also traced a couple of other myoblast-specific markers *viz. Pax3* and *MyoD1* and found that *Pax3* lineage was represented more broadly in the global brown adipocyte population, while *MyoD1^Cre^* did not trace any of the brown adipocyte population confirming that they do not arise from *MyoD1* lineage (26). Furthermore, some other studies have also reported the heterogeneous adipocyte labeling with *Myf5* lineage (28, 29). Altogether, these studies suggest that brown adipocytes from different depots or even within the same depots could derive from different embryonic lineages. Moreover, another recent report described that only 50% of adipocytes in iBAT derive from Pax7^+^ lineage originating from the central dermomyotome (30); which initially was thought to be a sole source of iBAT.

**Figure 1:**
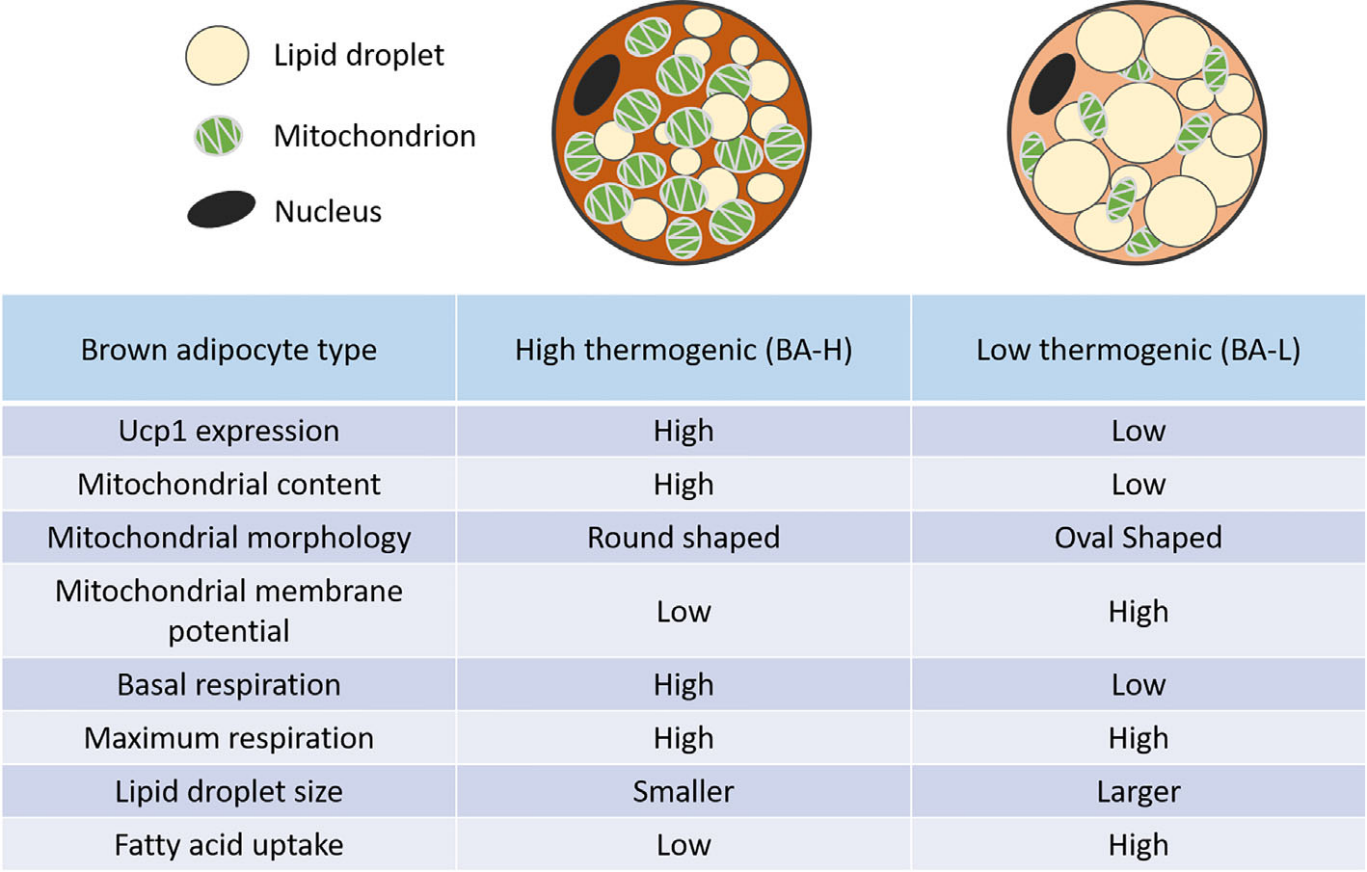
Distinct features of high and low thermogenic adipocytes. Relative to the high thermogenic brown adipocytes (BA-H), the low thermogenic brown adipocytes (BA-L) have lower UCP1 expression, low mitochondrial content, high mitochondrial membrane potential, and distinct mitochondrial morphology. These cells have a lower basal respiration rate, but a similar maximum respiration rate. They also have larger lipid droplets, and a higher fatty acid uptake rate (10).

Several groups have reported the heterogeneous UCP1 expression as well as mitochondrial potential in brown adipocytes (31–34). Spaethling et al. using a single-cell RNA sequencing (scRNA-seq) of nine handpicked mature brown adipocytes showed a transcriptome variability of brown adipocytes. In addition to variability in the expression of well-known brown adipocyte markers such as UCP1 and *Adrb3*, they also identified differential expression of various transporters as well as receptors for neurotransmitters, cytokines, and hormones (32). Similarly, recent work from our lab reported a high degree of functional diversity in the iBAT of mice. More precisely, using a scRNA-seq of mature brown adipocytes we identified two metabolically distinct brown adipocytes; high thermogenic (BA-H) and low thermogenic (BA-L). For thermogenic activity/function, we refer to the ability of the brown adipocyte to get activated to significantly increase substrates oxidization. BA-H is the classical thermogenic population with high UCP1 expression, high mitochondrial content as well as high respiration rate. On the contrary, a novel BA-L population had low UCP1 expression, low mitochondrial content, a respiratory rate that is intermediate between white adipocytes and BA-H, but similar respiration potential (**Figure 1**) (10). Further investigation of the lineage and metabolic functions of this newly identified novel BA-L population will help uncover new cellular mechanisms of thermogenic regulation in BAT. Interestingly, a very recent report, using single nucleus RNA-sequencing (snRNA-seq), identified a unique, rare subpopulation of regulatory brown adipocytes (35). This novel subpopulation that increases in abundance at higher environmental temperature modulates the thermogenic activity of neighboring adipocytes. The higher number of these cells in humans as compared to mice may explain the lower thermogenic capacity of human BAT. It is important to note here that none of these studies (except one by Bertholet et al.) including ours measured mitochondrial respiration in presence of GDP which represents a direct UCP1 dependent respiration. Seahorse Xfe mitochondrial stress test is utilized commonly by researchers to report the thermogenic or UCP1 function in brown adipocytes. However, it is not a direct measure of UCP1 activity. Although BA-L population has lower UCP1 protein level, it is not determined if these UCP1 protein has similar activity as UCP1 in the BA-H cells. Our future plan is to use GDP as a direct inhibitor of UCP1 in the mitochondrial stress test to measure UCP1 dependent thermogenic activity (36).

The biggest disadvantage of scRNA-seq is the possible alterations in gene expression because of dissociation and cell isolation procedures used while making single-cell suspensions. Although single-cell/nucleus sequencing technologies can dissect the cellular heterogeneity of the tissue at high resolution, the spatial information however is lost in the process. To help retain such information recent technologies such as spatial transcriptomics (37) and multiplexed *in situ* hybridization (38, 39), or visium spatial gene expression analysis (40) should be utilized along with sn/scRNA-seq. At the moment, even if spatial transcriptomics technologies do not provide a resolution at a single-cell level, progress is being made to achieve it by every passing day. Its application to frozen tissues is one important advantage though, making it a valuable resource for precious samples such as banked human biopsies. Lastly, integrating other omics technologies with sn/scRNA-seq to quantify proteins (41), cell surface epitopes (42) and chromatin accessibility (43) simultaneously will allow us a better understanding not only of the heterogeneity of these cells but also the cellular interactions present in the tissue. Finally, whether these distinct adipocyte types really represent distinct cell types or whether they are just the same cell types under distinct metabolic states remains to be further studied.

In humans, BAT was initially thought to exist only in infants to cope with the cold conditions during and after birth and eventually become metabolically inactive and disappear during adulthood. However, the presence of active BAT has been reported in outdoor workers from northern Finland as early as 1981 (44). Furthermore, there are several reports based on PET/CT scans of pheochromocytoma patients suggesting the presence of active BAT in adult humans (45–47). Later on other dedicated cold exposure as well as retrospective studies confirmed these observations (48–53). A recent report has defined several additional novel brown fat depots in mice, which are anatomically comparable and share molecular similarities with humans (54). An earlier study found the overlap between brown and beige molecular markers in human supraclavicular BAT (55). Later, the Kajimura group, using genetic profiling of clonally derived human brown adipocytes, elegantly showed that their molecular signatures were closely associated with those of mouse beige adipocytes (56, 57). This also led to the identification of human brown adipocyte molecular markers such as potassium channel K3 and mitochondrial tumor suppressor 1, which were found to be essential for beige adipogenesis in mice (56, 57). Using genetic profiling approach for preadipocytes derived from human neck fat, the Tseng group identified CD29 as a surface marker that specifically marks preadipocytes with high thermogenic potential (58). Furthermore, human beige adipocytes derive from the progenitors residing in the capillary network and have been shown to proliferate in response to pro-angiogenic factors (59). Interestingly, a recent study suggested that brown adipocytes, but not beige adipocytes of the physiologically humanized mouse had the thermogenic potential. They also found that the BAT of these mice closely resembles that of adult humans both morphologically as well as transcriptionally (60). It is important to note here that the difference between this study and the earlier study (57) is that the earlier study used young mice housed in standard conditions and were fed a normal chow diet. In the recent study by the Petrovic laboratory, they used physiologically humanized mice that are middle-aged, have been fed a high-fat diet, and are housed at thermoneutral temperatures. Lately, the housing temperature of mice for the metabolic studies has been questionable as standard conditions represent higher basal metabolic rate (BMR) in mice than that humans show at thermoneutrality. A recent report by Fischer et al. suggested housing mice at higher temperatures such as 30°C to achieve BMR comparable to resting humans (61), however as per Keijer et al. the best temperatures to achieve comparable BMR are between 25.5 – 27.6°C (62). So, it is important to consider housing temperatures and diets while planning and even comparing different mouse studies involving metabolic analyses. Lastly, the fact that only brown adipocytes and not the beige adipocytes of humanized mice retain thermogenic capacity, suggests the decreased thermocapacity of beige adipocytes during aging, thus making BAT an attractive target for therapeutics of metabolic disorders. Nevertheless, these data provide important insights into the heterogeneous nature of both rodent and human thermogenic adipocytes.

In summary, brown adipocytes within BAT represent high heterogeneity, and characterization of these distinct subpopulations will enable us to elucidate BAT thermogenic functions and regulations in detail. BAT’s heterogeneous nature offers new critical aspects to consider for future attempts that pursue novel therapeutic targets that activate BAT thermogenesis.

## Timeline of BAT Development

BAT depots appear earlier than WAT depots during embryogenesis (3). Also, most studies reporting such information have studied only a classic BAT depot iBAT. Early studies in rodents like mice and rats using mRNA measurements of mitochondrial markers such as cytochrome oxidase, UCP1, and ATP synthase found clusters of brown adipocytes appearing in the interscapular region around E15-16. UCP1 expression in these cells was hardly detectable during early embryogenesis and was found to be abruptly increased just before the birth around E18-19. This suggests the functional transformation of these cells into thermogenic competent brown adipocytes around E18-19 (63, 64). Another study used immunostaining of master regulator of adipogenesis, PPARγ (65, 66), to identify iBAT depots and found differentiating brown adipocytes as early as E14.5 (28). In a very recent report, in-situ hybridization of another critical factor involved in adipogenesis, C/EBPα (67, 68), demonstrated that the differentiation of adipocytes in iBAT starts at E12.5 and is functionally complete at E17.5 in the mouse embryo (69). Using the AdipoChaser mouse model based on adiponectin promoter (70), we recently suggested that the development of brown adipocytes in the iBAT starts as early as E10, and is most active at E14 (10). The generation of new brown adipocytes finishes by E16, as there were no new brown adipocytes labeled beyond this point. Furthermore, we showed that the heterogeneity in the iBAT cell population is achieved shortly after birth, around two weeks postnatally (10). Apart from the classic iBAT depot, several other BAT depots analogous to those observed in humans have been recently identified and characterized (54, 71). However, it remains unknown if they share a similar developmental timeline.

Postnatally, the BAT is a very plastic tissue and has been shown to undergo dramatic morphological alterations when exposed to different environmental temperatures. When exposed to thermoneutral temperature (30° C) as compared to standard housing conditions (22-24° C), brown adipocytes in mice undergo hypertrophic expansion with lipid droplets coalescing into a unilocular lipid droplet, leading to WAT like morphology as well gene expression profile. However, these cells maintain their molecular identity and are ready to go back to the classical morphology (72, 73). The Granneman group showed that the cold exposure at 4°C could induce brown adipogenesis in mice and their genetic lineage tracing model demonstrated that new adipocytes are derived from PDGFRα^+^ progenitors. Such *de novo* brown adipogenesis was restricted to the dorsal edge region of the iBAT (74). It remains unknown if long-term cold exposure can induce brown adipocyte recruitment in other BAT depots. Our work showed that cold exposure within a few days alters brown adipocytes’ heterogeneity, converting low-thermogenic brown adipocytes into high-thermogenic brown adipocytes (10). Both obesity and aging have been associated with the reduction of BAT thermogenic capacity (75, 76). Interestingly, the phenomenon of dynamic interconversion of BA-L and BA-H was not affected by the high-fat diet (HFD) feeding, but it declined with age (10). These observations provide important insights into the mechanisms of BAT thermoregulation and might help explaining its reduced thermogenic capacity during aging. It will be interesting to investigate the molecular mechanisms that regulate and balance the equilibrium between *de novo* brown adipogenesis and interconversion of high and low thermogenic brown adipocytes after cold exposure.

## Non-Shivering Thermogenesis in BAT

Adaptive thermogenesis is the most important metabolic function of BAT. There are two types of adaptive thermogenesis: cold-induced thermogenesis (77) and diet-induced thermogenesis (78). Skeletal muscle-based shivering accounts for a portion of cold-induced thermogenesis. However, with the cold adaptation non-shivering thermogenesis in BAT becomes prominent (79). The primary mechanism of BAT non-shivering thermogenesis involves the uncoupling of the mitochondrial respiratory chain, which is mediated *via* UCP1; a proton transporter located on the inner mitochondrial membrane (8, 9, 80–82). This uncoupling results in increased substrate oxidation and dissipation of energy in the form of heat (83, 84). The rich vascularity of BAT helps in redistributing the generated heat across the body for temperature homeostasis (84). BAT is also highly innervated by sympathetic neurons (85). During cold exposure, activation of β3-adrenergic receptors by norepinephrine leads to lipid catabolism liberating free fatty acids and also induces the expression of UCP1 as well as other pro-thermogenic genes (3, 86). Apart from catecholamines some other non-sympathetic molecules such as triiodothyronine (T3), hepatic bile acids, and various retinoids have also been shown to play a role in the thermogenic activation of brown adipocytes (3). Classical studies performed using isolated BAT mitochondria have suggested an essential role of fatty acids as a regulator of brown adipocyte mitochondrial respiration (87, 88). A recent study using a patch-clamp measurement of UCP1 currents in BAT mitochondria found that fatty acids serve as an anion transporter to transport protons across the inner mitochondrial membrane. Both short-chain fatty acids and long-chain fatty acids are associated with UCP1. However, long-chain fatty acids are unable to dissociate because of hydrophobic interaction (89). Moreover, cold exposure has been shown to increase the activities of several antioxidant enzymes such as superoxide dismutase, catalase, glutathione peroxidases, and glutathione reductase in rat BAT suggesting elevated oxidative stress in BAT during thermogenesis (90).

Interestingly, several recent studies revealed that elevating the levels of reactive oxygen species (ROS), using genetic or pharmacological approaches, increased whole-body energy expenditure and protected against diet-induced obesity, along with increased brown adipocyte mitochondrial respiration (91–94). These observations suggest a critical role of elevated ROS in the thermogenic regulation of brown adipocytes. Furthermore, Cys253 of UCP1 has been recently shown to be sulfenylated by increased mitochondrial ROS in activated brown adipocytes, and this modification was found to be an important regulatory mechanism of UCP1-dependent thermogenesis (95). Moreover, succinylation of lysine residues (K56 and K151) of UCP1 by Sirtuin 5 has been suggested to modulate its stability and activity (96). When there is no thermal stress, UCP1 is usually thought to be functionally inhibited by purine nucleotides (97, 98). A recent report showed that the activation of brown adipocytes by adrenergic stimulation resulted in the degradation of these purine nucleotides in brown adipocytes both *in vivo* and *in vitro*, leading to the activation of UCP1 (99). These observations emphasize an essential role of UCP1 in BAT thermogenesis and further understanding of the regulation of its activity might uncover novel mechanisms of thermoregulation.

Genetic ablation of UCP1 in mice led to fatal hypothermia after cold exposure (100). Interestingly, the cold sensitivity of these mice was dependent on the genetic background. Mice with congenic C57BL/6J and 129/SvImJ backgrounds were cold-sensitive, whereas those on F1 hybrid background were found to be resistant to cold (101). Also, the effects of diet-induced obesity on these mice were found to be temperature-dependent (102, 103). Moreover, when UCP1-null mice were gradually exposed to reduced environmental temperatures, they could regain their ability to acclimatize to cold (104). These findings suggest that UCP1-independent mechanisms of thermogenesis may exist. As an alternate thermogenic mechanism, creatine cycling was initially discovered in the murine beige adipocyte mitochondria (34, 105). Ablation of the creatine synthesis rate-limiting enzyme glycine amidinotransferase in an adipocyte-specific manner in mice resulted in reduced BAT creatine concentration and mild cold intolerance (106). Recently, creatine kinase B (CKB), as the only isoenzyme in brown adipocytes, is proven to be indispensable for the futile creatine cycle-related thermogenesis (107). These studies revealed the critical role of creatine cycling as an alternate thermoregulatory mechanism in brown adipocytes.

## Brown Adipocyte Energy Metabolism

As BAT has exceptionally high energy expenditure, it is not surprising that it is an essential player in whole-body metabolic regulation. As mentioned above, studies done using BAT transplantation approaches suggested a major role of BAT in glucose homeostasis in mice (108–110). Similarly, activating BAT by cold exposure in humans led to improved whole-body glucose homeostasis and insulin sensitivity in both healthy individuals and individuals with type 2 diabetes (111, 112). With Seahorse Analyzers, for the first time, we were able to measure oxygen consumption in primary mature brown adipocytes, which is largely different from BAT tissue chucks, or *in vitro* cultured brown adipocyte differentiated from primary stromal vascular fraction (10). These primary mature brown adipocytes had 5-10 folds higher basal respiration, per cell, compared to the cells in the stromal vascular fraction of the same BAT, or mature white adipocytes from the same mouse (10). This result further validated that brown adipocytes would utilize energy at a much higher rate than other cell types, even at the basal, unstimulated status.

Although BAT is known to have a high rate of glucose uptake, fatty acids are commonly viewed as the primary fuel for mitochondrial uncoupling respiration (49, 51, 52, 54, 113–118). The Jiang group’s most recent work used *in vivo* [U-^13^C]glucose tracing and demonstrated that BAT activation by chronic cold exposure leads to increased glucose oxidation and enhances glucose flux to mitochondrial tricarboxylic acid cycle (119). Such increase in glucose uptake by adrenergic stimulation is, however, found to be independent of UCP1 presence or activity (120). Moreover, BAT has been shown to express pyruvate carboxylase; which can further enhance the glucose uptake by promoting anaplerosis (121). Mitochondrial pyruvate carrier (MPC) connects cytosolic glycolysis and mitochondrial glucose oxidation (122, 123). Most importantly, inhibition of MPC in mice resulted in blockade of cold-induced glucose oxidation in BAT, thereby impairing the body temperature homeostasis (119). In line with this, another group also showed that BAT-selective ablation of mitochondrial pyruvate carrier 1 (MPC1) in mice led to impaired cold sensitivity and glucose handling (124). Moreover, MPC inhibition in *in vitro* differentiated brown adipocytes, without any adrenergic stimulation, resulted in increased mitochondrial fatty acid oxidation and lipid cycling; thereby increasing the energy expenditure (125). Thus, limiting pyruvate uptake in brown adipocyte mitochondria could be an effective way of increasing the energy expenditure in the absence of adrenergic stimulation. These observations point toward an important role of glucose oxidation in BAT thermogenesis.

Fatty acids and lipolytic agents have been shown to stimulate respiration in brown adipocytes (126). Stimulation of β3-adrenoreceptor results in increased uptake as well as utilization of FFAs in BAT, but not in WAT (127). BAT uses circulating FFAs after hydrolysis of triacylglycerol-rich lipoproteins (TRLs) as a substrate for thermogenesis (128). Notably, lipoprotein lipase (LPL), an enzyme that is required for this hydrolysis, was induced several folds in BAT during cold acclimatization (129). Moreover, this induction has been shown to contribute to vascular lipoprotein homeostasis during cold exposure by channeling TRLs to BAT (130). This effect can also be partly attributed to the downregulation of a secreted protein angiopoietin-like 4 which inhibits LPL activity. This downregulation further potentiates the LPL activity, thereby increasing the uptake of TRLs in BAT, leading to increased systemic triglyceride clearance (131). Intriguingly, BAT volume during thermoneutral or cold exposures was found to be positively correlated to lipolysis, FFA cycling as well as oxidation, and adipose insulin sensitivity in humans (132). Global deletion of adipose triglyceride lipase (ATGL); a rate-limiting enzyme involved in lipolysis of lipid droplet triglycerides, resulted in defective cold adaptation in mice. This suggested an essential role of ATGL in fueling thermogenesis by locally derived FFAs (133). Indeed, adipocyte-specific ablation of ATGL in mice led to the conversion of BAT to WAT-like tissue and resulted in severely impaired thermogenesis (134). However, recent studies using a BAT-specific inhibition of lipolysis using genetic approaches suggested that BAT lipolysis is not essential for cold-induced thermogenesis (135, 136). Cold intolerance previously observed in global ATGL KO mice was attributed to the impaired cardiac function. Another recent study that impaired the triglyceride synthesis and storage in the BAT lipid droplets by BAT-specific deletion of triglyceride synthesis enzymes also suggested that BAT lipid droplets are dispensable for cold-induced thermogenesis (137). While there is no doubt that BAT could utilize a large amount of glucose and fatty acids, it would be fascinating to explore further how brown adipocyte selects the primary fuel for thermogenesis, and how this selection would have a dynamic impact on whole-body glucose and lipid homeostasis.

**Figure 2:**
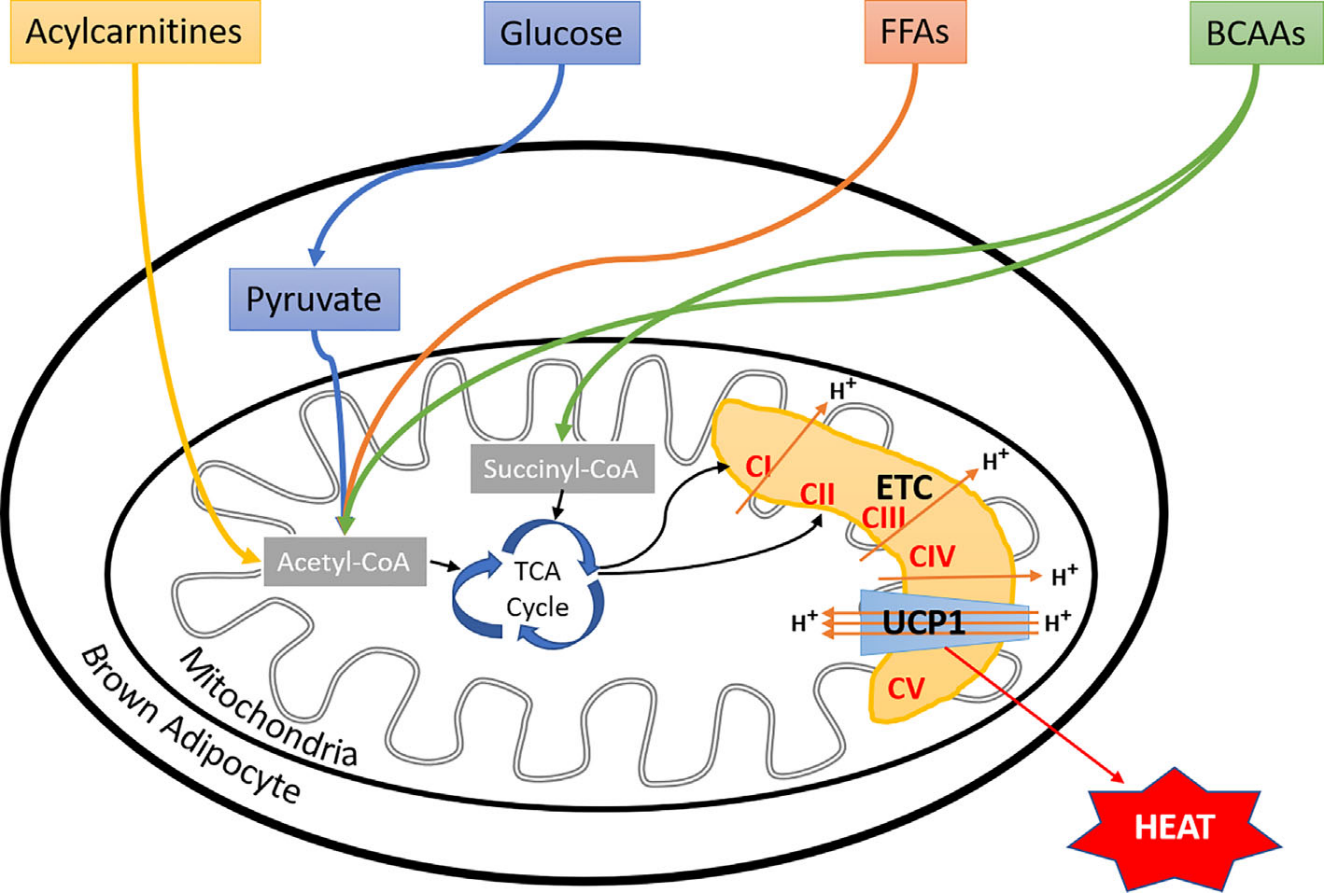
Fuel selection by brown adipocyte. Besides glucose and fatty acids, BAT utilizes a variety of substrates, including BCAAs, succinate, and liver-derived acylcarnitines to fuel thermogenesis, more substrates to be discovered in the near future. However, the regulatory mechanisms of brown adipocyte fuel selection, especially upon environmental temperature changes, and whether aging or metabolic disorders affect these processes remain unknown. FFAs, free fatty acids; BCAAs, branched-chain amino acids; ETC, electron transport chain; CI, complex I; CII, complex II; CIII, complex III; CIV, complex IV; CV, complex V; UCP1, uncoupling protein I.

Apart from FFAs and glucose, branched-chain amino acids (BCAAs) have also been shown to fuel BAT thermogenesis in mice and humans (138). Interestingly, increased blood levels and impaired metabolism of BCAAs, have been linked to the etiology of type 2 diabetes (139, 140). These observations may provide a novel link between impaired BAT thermogenesis and metabolic disorders. Furthermore, the Kajimura group suggested that stimulation of BCAA catabolism by activating BAT may protect against insulin resistance development by preventing the activation of mTOR signaling (138). Moreover, ^13^C-labeled isotope tracing of preadipocytes and differentiated adipocytes showed increased BCAA catabolism in differentiated adipocytes as compared to proliferating cells, which used glucose and glutamine to fuel the mitochondrial oxidation. Inhibition of BCAA catabolism resulted in impaired adipogenesis, suggesting an important functional role of BCAAs in adipocyte differentiation (141). Additionally, the Chouchani group found a significant accumulation of succinate (92), a tricarboxylic acid cycle intermediates in cold-activated brown adipocytes, which was independent of adrenergic signaling. Interestingly, the administration of succinate to mice also led to the activation of BAT thermogenesis. Mechanistically this effect was dependent on succinate dehydrogenase generated ROS (92). Another recent report showed that FFAs, released from white adipocytes in response to cold exposure, induced hepatocyte nuclear factor 4 alpha mediated acylcarnitine production in the liver (142). This led to increased plasma concentration of acylcarnitines, which were taken up by BAT to fuel the thermogenesis. Most importantly, supplementation with L-carnitine or palmitoylcarnitine rescued the age-dependent cold sensitivity in mice, suggesting an essential role of acylcarnitine metabolism in age-induced impairment of thermogenesis (142). Altogether, brown adipocytes utilize multiple substrates as fuels for thermogenesis (**Figure 2**). Again, how brown adipocytes perform fuel selection among all these substrates remains mostly unknown, and yet it is unclear if there are switches of fuel selections during aging or the development of metabolic disorders.

Lastly, as mentioned earlier, it is important to perform BAT metabolic studies in rodents at thermoneutral temperatures to generate data that is comparable to humans. Although studies in humans have confirmed the presence of thermoactive BAT, one should note that the prevalence of BAT was increased only after cold exposure. In warm conditions little to no BAT was detected in these subjects (50–52). Moreover, overweight, and obese subjects showed significantly lower BAT activity (52). Also, such cold-induced BAT activation was higher during winter as compared to summer (50). Furthermore, Yoneshiro et al. showed that the cold-induced thermoactivation of BAT reduced during aging; as the incidence of cold-activated BAT fell from about 50% in the twenties to less than 10% in the fifties and sixties (75). So, taken together factors such as temperature, age, and dietary compositions should be carefully considered while performing metabolic studies related to BAT or as a matter of fact related to any other metabolically active organ in both rodents and humans.

## BAT as an Endocrine Organ

The WAT is well established as an endocrine organ, secreting adipokines such as adiponectin (143) and leptin (144). There is recently ample evidence supporting the fact that BAT may act as a unique endocrine organ by secreting some factors, so-referred to as “batokines” (145). In the 1980s, *Silva and Larsen* found that the enzyme type 2 iodothyronine deiodinase (DIO2) is specifically expressed in BAT, and it converts thyroxine (T4) to triiodothyronine (T3) (146). They also showed that its activity is strongly induced during thermogenesis, and BAT serves as an important site for both local and systemic T3 generation (147). Both DIO2 and T3 have essential functions in regulating BAT thermogenesis (148, 149). Fibroblast growth factor-21 (FGF21), an essential player in glucose oxidation in multiple organs, was found to be upregulated in BAT in response to cold exposure as well as adrenergic stimulation (150, 151). Furthermore, cytokine interleukin-6 (IL-6) is induced during thermogenesis in mouse brown adipocytes (152). BAT from healthy mice, when implanted in HFD fed mice, improved glucose homeostasis and insulin sensitivity. This effect was found to be mediated *via* endocrine actions of IL-6 as BAT implantation from IL-6 KO mice failed to show such improvements (108). Likewise, the insulin-independent reversal of type I diabetes (T1D) was achieved when BAT from healthy mice was transplanted in the streptozotocin-induced diabetic mouse model. Such transplantation, if done before the induction, was even able to prevent or significantly delay the development of T1D. This antidiabetic effect of BAT implantation was attributed to insulin-like growth factor-1 (IGF-1), which was upregulated in the tissue transplants. It is supposed to mediate its effects *via* improving the WAT inflammation, adipogenesis, and direct effect on insulin receptors (109, 110). Like many other cell types, brown adipocytes also secrete Vascular endothelial growth factor-A (VEGF-A), a signaling protein that promotes the growth of new blood vessels. VEGF-A is essential for the activation and expansion of BAT (153). Importantly, another brown adipocyte enriched factor neuregulin 4 (*Nrg4*) has been demonstrated by the Lin laboratory to protect against diet-induced insulin resistance as well as hepatic steatosis in mice. This is achieved by negatively regulating the *de novo* lipogenesis in the liver and by activating hepatic fatty acid oxidation (154, 155). *Nrg4* transgenic mice also showed increased energy expenditure and improvement of whole-body glucose metabolism (154, 155). The Kahn laboratory recently discovered BAT-derived circulating miRNAs, which control the gene expression in the liver, especially that of FGF21. Mice lacking miRNA processing enzyme, Dicer, specifically in adipose tissue, had improved glucose tolerance (156). Furthermore, some lipid-derived lipokines, such as 12,13-dihydroxy-9Z-octadecenoic acid (12,13-diHOME) and 12-hydroxyeicosapentaenoic acid (12-HEPE) are secreted specifically by BAT. 12,13-diHOME promotes fatty acid uptake in skeletal muscle and BAT, leading to enhanced cold tolerance and improved systemic triglyceride clearing (157, 158). 12-HEPE, on the other hand, improved the whole-body glucose homeostasis by increasing glucose uptake in skeletal muscle and adipocytes (159). Lastly, secretome analyses of brown adipocytes using modern-day proteomics and transcriptomics approaches have identified several novel batokine candidates including ependymin-related protein 1 (EPDR1) and phospholipid transfer protein (PLTP) (160–162). EPDR1 was found to be an important commitment factor for brown adipogenesis (161). Whereas, PLTP improved glucose and lipid homeostasis *via* the regulation of liver lipoproteins and bile acids (160). Lastly, recruitment of immune cells in BAT; especially that of activated macrophages has been shown to be associated with the thermogenic activation (163). Chemokine C-X-C motif chemokine ligand-14 (CXCL14) is another example of batokine secreted by BAT in response to adrenergic stimulation and has been shown to play an important role in activation and recruitment of macrophages to BAT during thermogenic activation (164). Taken together, these recent studies highlighted the function of BAT as a unique endocrine organ, playing essential functions in regulating whole-body metabolic homeostasis.

## BAT Centered Therapeutic Approaches and Future Perspectives

BAT plays an essential role in energy homeostasis. Upon activation, BAT can function as an effective energy sink, burning and disposing excess lipids and glucose. Unfortunately, BAT activity declines during aging or the development of metabolic disorders (49, 75, 165). Therefore, enhancing BAT thermogenic activity has been an attractive strategy for the treatment of obesity and type 2 diabetes. Indeed, thermogenic activation of BAT either by cold exposure (111, 113, 166, 167) or by adrenergic stimulation *via* β3-adrenoreceptor (AR) agonist Mirabegron (168) showed beneficial metabolic effects such as increased BAT glucose uptake, improved insulin sensitivity, and weight loss in humans. Furthermore, several synthetic molecules acting *via* different mechanisms have recently been shown to activate BAT in mice, increasing whole-body energy expenditure (169–172). The clinical applications of these compounds are being actively evaluated. Although activating BAT might seem an exciting target for treating metabolic disorders, it is worth noting that humans have various responses to the same stimulation regarding BAT activity. BAT mass is negatively correlated with age as well as diabetic status (173), making this approach more challenging in aged as well as diabetic individuals. BAT transplantation studies in mice have shown the vital role of BAT in the regulation of adiposity, glucose homeostasis, and insulin resistance (108–110, 174, 175). Interestingly, brown adipocytes engineered from human fibroblasts or stem cells from human WAT stromal vascular fraction were transplanted in mice in multiple studies (176–178). In general, these transplantations showed beneficial metabolic effects, such as protection from diet-induced adiposity and insulin resistance. A similar approach can be used in humans, in theory, to increase functional BAT mass. Moreover, common dietary supplements such as L-arginine and capsinoids have been shown to increase BAT recruitment and activation, leading to beneficial effects with respect to glucose homeostasis and insulin sensitivity in both mice and humans (179–181). Lastly, as mentioned above several secretory factors having endocrine functions have been recently identified from BAT. These batokines may be considered emerging therapeutic targets for metabolic disorders. For instance, a recent report by Baruch et al. showed that FGF21 mimetic antibody BFKB8488A when injected subcutaneously in overweight/obese human subjects, resulted in a reduction in body weight, improved cardiometabolic parameters, and reduced carbohydrate intake (182). Taking together, the rediscovery of functional BAT depots in humans has undoubtedly sparked a new era of research about the therapeutic targeting of this tissue for its amazing metabolic health benefits. However, a detailed understanding of the basic biology of its development, heterogeneity, and metabolic regulation will surely further aid this cause.

## Author Contributions

ABS, QW, and AS wrote the manuscript. All authors contributed to the article and approved the submitted version.

## Funding

QAW was supported by US National Institutes of Health grants R01AG063854, R01HD096152, American Diabetes Association Junior Faculty Development Award 1-19-JDF-023, and City of Hope Caltech-COH Initiative Award.

## Conflict of Interest

The authors declare that the review was construed in the absence of any commercial or financial relationships that could be constructed as a potential conflict of interest.
